# Analytical cohort study on cognitive reserve as a predictor of post-stroke dementia severity

**DOI:** 10.6026/973206300213480

**Published:** 2025-10-31

**Authors:** Shreya Krishna, Subash Kumar, Elakkiya L., Shanmukha Koppolu, Shabrin Abdul Rasheed, Vijaya Madhuri Devi Kunche, Ayush Bhardwaj

**Affiliations:** 1 Department of Medicine, Nottingham University Hospitals NHS Foundation Trust, Nottinghamshire, United Kingdom; 2 General Practitioner, General Medicine, Madras Medical College, Chennai, India; 3 Department of General Medicine, Madras Medical College, Chennai, India; 4 Department of Trauma and Orthopaedics, Barts NHS Trust, London, England, United Kingdom; 5 Department of Emergency Medicine, Basildon University Hospital, Nethermayne, Basildon, Essex, SS16 5NL, United Kingdom; 6 Department of Critical Care Medicine, Dr. NTR University of Health Sciences, Vijayawada, Andhra Pradesh, India; 7 Internal Medicine, Manipal Academy of Higher Education (MAHE), Karnataka, India

**Keywords:** Cognitive reserve, post-stroke dementia, stroke survivors, dementia severity, neuroprotection, cognitive aging, education, occupational complexity, longitudinal analysis, neuroplasticity

## Abstract

The role of cognitive reserve in determining the severity of dementia following a stroke in individuals aged 55 and above is of interest. Hence, a total of 128 stroke survivors were followed for 12 months post-event, with cognitive reserve quantified using education level, occupational attainment and engagement in cognitive activities. Higher cognitive reserve scores were associated with milder post-stroke dementia outcomes and better functional independence. Thus, we show that cognitive reserve is a significant protective factor against severe cognitive decline after stroke.

## Background:

Post-stroke dementia (PSD) is a common and disabling outcome; it affects approximately one out of three stroke survivors and significantly affects individuals' independence, daily functioning and quality of life overall [1]. Predictors of PSD include older age, worse stroke severity, recurrent vascular events and the presence of comorbid conditions, such as hypertension, diabetes and atrial fibrillation [2]. However, biological and vascular predictors do not fully explain cognitive trajectories in stroke patients. An emerging construct that might explain these varying trajectories is cognitive reserve (CR), which is defined as the mind's ability to resist neuropathological damage through efficient or flexible or cognitive processing strategies [3]. Cognitive reserve is thought to accrue across the life course through experiences such as intellectual enrichment, educational attainment, occupational complexity and engaging in cognitively challenging and stimulating leisure opportunities and activities [4]. It is thought people with higher cognitive reserve can choose more flexible neural networks or pathways which reduce cognitive impairment to brain injuries of equivalent degree and severity [5]. The protective function of cognitive reserve (CR) has been established in Alzheimer's disease and other neurodegenerative dementias, with associations with a delay in the onset of symptoms and with slower clinical progression [6]. In contrast, there are much fewer studies in post-stroke dementia (PSD) and other vascular cognitive impairment conditions and the findings here, while interesting, are far more mixed [7]. Some studies suggest that higher education levels and more mental activity provide a buffer against cognitive decline after stroke, while others have identified relatively small or inconsistent effects. Considering that ischemic and hemorrhagic strokes disrupt neural networks that support memory, attention and executive function, examining the influence of prior CR on post-stroke cognitive outcomes should be of clinical interest [8]. Understanding the role of CR in post-stroke cognitive outcomes could inform better risk stratification models, assist in patient and family counseling and inform rehabilitation programs designed to enhance the cognitive resilience of patients. Therefore, it is of interest to report the predictive value of cognitive reserve on the severity of dementia following stroke, using a multidimensional CR index incorporating education, occupational complexity and cognitive leisure activities.

## Materials and Methods:

This prospective analytical cohort study was conducted at two tertiary stroke centers between January 2022 and April 2024. A total of 128 first-time ischemic stroke survivors aged 55 years and older were enrolled consecutively after hospital discharge. Inclusion criteria included confirmed diagnosis of ischemic stroke via MRI/CT, ability to participate in cognitive assessments within 3 months post-stroke and no prior clinical diagnosis of dementia or major psychiatric illness. Patients with severe aphasia or sensory deficits impeding cognitive testing were excluded. Cognitive reserve was measured using a validated composite CR index derived from three domains: (1) educational attainment (years of formal education), (2) occupational complexity (classified using the International Standard Classification of Occupations) and (3) cognitive leisure activity score, which assessed frequency of reading, writing, puzzles and social engagement over the preceding 10 years. Each domain was scored on a scale of 0 to 4, with a maximum CR index score of 12. Participants underwent baseline assessments within 12 weeks of stroke, including neurological examination (NIH Stroke Scale), neuroimaging and initial cognitive testing using the Montreal Cognitive Assessment (MoCA). Functional independence was evaluated using the modified Rankin Scale (mRS). Follow-up cognitive assessments were performed at 12 months post-stroke using Clinical Dementia Rating (CDR) and standardized neuropsychological batteries to classify dementia severity into mild, moderate, or severe. Data were analyzed using SPSS version 27. Descriptive statistics were used for demographic and clinical variables. Multivariate logistic regression models were used to assess the association between CR index and dementia severity while adjusting for confounders such as age, stroke volume, hypertension, diabetes and education. A p-value < 0.05 was considered statistically significant. Ethical clearance was obtained from both institutional review boards and informed consent was provided by all participants or their caregivers.

## Results:

Cognitive reserve (CR) was also identified as an important predictor of post-stroke dementia (PSD) outcomes at one-year follow-up amongst the 128 stroke survivors. Participants with higher mean CR scores were more likely to report experiencing mild PSD. In contrast, participants with low mean CR scores frequently progressed from mild dementia to moderate or severe dementia. Education and occupational complexity contributed to higher CR independently and participants with advanced education or cognitive jobs had significantly better rates of moderate or severe PSD. Similar to CR, those who reported frequent engagement in cognitive leisure activities appeared to have some level of protection against dementia and no participants who reported the highest level of cognitive activity engaged experienced severe dementia. Overall functional independence, as measured with the modified Rankin Scale, was significantly associated with higher CR scores, indicating both cognitive and physical recovery. Severity of the stroke was significantly associated with the dementia outcomes at one-year follow-up; however, CR continued to moderate the relationship and minimized poor dementia outcomes, even for individuals with severe strokes. Regression analysis indicated a strong and independent association with severe PSD outcomes along with stroke severity, but other factors such as older age and diabetes had weaker or non-significant associations. Gender was not significantly associated with CR scores or the dementia outcomes. Participants who have higher levels of education, a more diverse occupational history and more engagement in cognitive related leisure activities were less likely to develop moderate or severe levels of dementia. Overall, these results help to further support cognitive reserve as a strong, independent variable in predicting PSD progression while also acting as a mediator for demographic, educational, occupational and clinical variables. Table 1 (see PDF) shows a relatively even spread across categories, with the majority clustered in the moderate CR range. Table 2 (see PDF) shows Post-stroke dementia severity by CR level shows that severe dementia was concentrated among those with low CR, whereas mild dementia predominated in participants with high CR. Table 3 (see PDF) shows Education level and dementia severity shows that higher educational attainment was associated with milder dementia outcomes, with severe dementia most frequent among those with ≤6 years of education. Table 4 (see PDF) shows Occupational complexity and dementia severity shows those individuals with cognitively demanding professional roles had the lowest rates of severe dementia compared to that in manual labor. Table 5 (see PDF) shows Cognitive activity score versus dementia severity shows that frequent participation in leisure activities correlated with milder PSD, with no severe dementia among the highest activity group. Table 6 (see PDF) shows Modified Rankin Scale (mRS) scores by CR level shows that functional independence at 12 months was highest among those with high CR scores. Table 7 (see PDF) shows Stroke severity (NIHSS) versus dementia severity shows that severe strokes were strongly linked with severe dementia, though higher CR still mitigated the risk. Table 8 (see PDF) shows Logistic regression predictors of severe PSD shows that low CR and severe stroke independently predicted severe dementia, with CR exerting a strong protective effect. Table 9 (see PDF) shows CR score distribution by gender shows no significant difference in CR scores between male and female participants. Table 10 (see PDF) shows CDR scores stratified by stroke severity and CR level shows that within each stroke severity group, higher CR was consistently associated with lower dementia ratings.

## Discussion:

The findings of this analytical cohort study strongly support the hypothesis that cognitive reserve (CR) serves as a protective factor against severe post-stroke dementia (PSD). Participants with higher CR scores-those with greater educational attainment, cognitively demanding occupations and regular engagement in cognitive leisure activities-consistently exhibited milder cognitive decline 12 months after stroke. This reinforces the notion that cognitive reserve buffers the brain against the deleterious effects of cerebrovascular insults, likely by enhancing neural efficiency, compensatory network recruitment and cognitive flexibility [9]. Educational attainment was one of the most potent contributors to higher CR and milder PSD outcomes, in line with previous literature suggesting that formal education facilitates lifelong neuroplasticity. Similarly, occupational complexity and sustained cognitive engagement appear to enhance the brain's adaptability to damage, which may explain the significantly lower Clinical Dementia Rating (CDR) scores observed in participants from professional or intellectually demanding fields [10]. These protective effects persisted even after adjusting for conventional risk factors such as age, diabetes and stroke severity, highlighting the independent predictive value of CR. Importantly, although stroke severity (as measured by NIHSS) was a major determinant of dementia progression, individuals with high CR scores consistently fared better even in the face of moderate to severe strokes [11]. This suggests that cognitive reserve may help preserve higher-order cognitive functioning despite substantial neurological damage. Furthermore, functional independence (as measured by the modified Rankin Scale) was significantly better in high-CR individuals, emphasizing not only a cognitive but also a broader rehabilitative advantage conferred by CR. Contrary to some expectations, no significant difference in CR was found between male and female participants, indicating that gender may not modulate cognitive resilience in this context when other contributing factors are accounted for [12]. However, lifestyle-related variables, including frequency of cognitive leisure activities, emerged as modifiable contributors to CR, providing a potential avenue for post-stroke rehabilitation strategies aimed at mitigating long-term cognitive decline these findings underscore the value of incorporating cognitive reserve assessment into routine stroke management and post-stroke prognostic models. They also highlight a unique opportunity for preventive intervention: promoting cognitive enrichment throughout life may not only delay the onset of dementia but also attenuate its severity when neurological injury occurs. Future longitudinal studies with larger, more diverse populations could further validate these associations and explore the underlying neurobiological mechanisms linking cognitive reserve and post-stroke outcomes.

## Conclusion:

Higher cognitive reserve significantly predicts milder post-stroke dementia severity, independent of age and stroke severity. Educational background, occupational complexity and cognitive engagement collectively enhance neurocognitive resilience. Integrating cognitive reserve assessment into stroke care may improve prognostic accuracy and guide personalized rehabilitation strategies.
